# Impaired graft survival in kidney transplants from expanded criteria donors with HLA-DR mismatch

**DOI:** 10.3389/frtra.2026.1733351

**Published:** 2026-02-12

**Authors:** Uwe Scheuermann, Philipp Rhode, Claudia Lehmann, Jan Kowald, Sophie Seiffer, Fabian Haak, Daniel Seehofer, Sebastian Murad Rabe, Hans-Michael Tautenhahn

**Affiliations:** 1 Department of Visceral, Transplantation, Vascular and Thoracic Surgery, University Hospital of Leipzig, Leipzig, Germany; 2 HLA Laboratory, Institute for Transfusion Medicine, University Hospital Hamburg-Eppendorf, Hamburg, Germany; 3 Medical Department III, Division of Nephrology, University of Leipzig Medical Center, Leipzig, Germany

**Keywords:** expanded criteria donor, HLA-DR, kidney transplantation, mismatch, outcome, survival

## Abstract

Human leukocyte antigen (HLA)-DR mismatches are known to increase the risk of graft rejection following kidney transplantation (KT). Especially in KT using grafts of lower quality from expanded criteria donors (ECD), the immune response plays a critical role. Therefore, the aim of this study was to investigate the impact of HLA-DR mismatches on both short- and long-term outcomes in primary ECD KT. 537 KT recipients were stratified into four groups based on their HLA-DR mismatch status [absent (−) or present (+)] and donor type [standard criteria donor (SCD) or ECD]: (1) SCD-DR-(*N* = 126), (2) SCD-DR+ (*N* = 167), (3) ECD-DR- (*N* = 60) and (4) ECD-DR+ (*N* = 184). Clinicopathological characteristics, transplant outcomes and survival were evaluated across the groups. Rejection-free graft survival was significantly decreased in recipients of ECD-DR + KT (*P* = 0.005). In comparison with ECD-DR- KTs, ECD-DR + KTs were associated with a significantly reduced graft survival (15.1 vs. 10.7 years; *P* = 0.034). After adjusting for relevant cofactors using multivariate Cox regression analysis, HLA-DR mismatch remained an independent predictor of graft rejection and graft survival in ECD kidney grafts. These results suggest that HLA-DR compatibility should be considered in allocation protocols for this donor category to improve long-term outcomes.

## Introduction

The persistent gap between the demand for and supply of deceased donor kidneys has led to an increased use of organs from expanded criteria donors (ECD). As defined in the literature, ECDs include all deceased kidney donors aged over 60 years, irrespective of the cause of death. Donors aged 50–59 are also classified as ECDs if they present with at least two of the following: a history of hypertension, a cerebrovascular cause of death, or a serum creatinine level greater than 1.5 mg/dL (1, 2). Although kidney transplantations (KT) from ECDs have been shown to increase transplant opportunities for older and comorbid patients and significantly extending recipientś life expectancy and quality of life compared to chronic dialysis, these organs are more frequently associated with delayed graft function and graft loss compared to standard criteria donor (SCD) organs (3).

In this context, the role of immunologic compatibility becomes increasingly important. Mismatches in human leukocyte antigens (HLA) between donor and recipient are well-established contributors to immune-mediated graft injury. In particular, mismatches in the Class II HLA molecule HLA-DR have been associated with poorer graft outcomes (4–6).

Although the impact of HLA-DR mismatches has been extensively studied, the individual interaction between immunological compatibility and donor kidney function is less well understood. In living donor kidney transplantation, higher donor eGFR significantly mitigates the risk of graft loss in recipients with HLA-DR mismatches, whereas no comparable benefit is observed in HLA-DR–matched recipients (7). And a previous study within the European Senior Program (ESP)—a program facilitating KT between older donors and recipients, typically aged 65 years and above—found that HLA-DR mismatches significantly increased the five-year risk of graft loss and mortality (8).

The clinical significance of HLA-DR mismatches in ECD transplant settings has not yet been investigated. This study therefore aims to assess postoperative outcomes, rejection rates and graft and patient survival in primary ECD KT in relation to HLA-DR matching.

## Patients and methods

### Data collection and study population

Medical data from all adult patients (≥18 years of age) who underwent initial deceased donor KT at the University Hospital of Leipzig between February 1994 and October 2024 were retrospectively analyzed. Our data source comprised a prospectively collected electronic database. Exclusion criteria were multi-organ (combined) transplants, re-transplants, missing data, kidney grafts from donors younger than 16 years, and graft loss due to technical failure within the first week after KT. Furthermore, to mitigate the confounding effects of preformed donor-specific antibodies, we excluded KT recipients with a pre-transplant PRA level greater than 5%. Follow-up data were collected until September 2025.

The characteristics of the study population included donor and recipient age, gender, body mass index (BMI; weight in kg/ height in m^2^), comorbidities, and preoperative cytomegalovirus status; donor cause of death, cause of recipient ESRD, duration of dialysis prior to KT. The estimated glomerular filtration rate was calculated with the Chronic Kidney Disease Epidemiology Collaboration equation and adjusted to the individual patient standard body surface area, as previously described (9).

Kidney donors were categorized into Standard Criteria donors (SCD) and Expanded Criteria Donors (ECD) according to the UNOS definition. Donors under 50 years of age were considered SCDs, those over 60 years as ECDs. Donors aged 50–59 were classified as ECDs if at least two of the following applied: history of hypertension, non-traumatic cerebrovascular cause of death, or serum creatinine >1.5 mg/dL; otherwise, they were considered SCDs (1, 2).

Transplant data included information on the number of human leukocyte antigen (HLA) -A, -B, and -DR mismatches (0–6), pretransplant PRA levels, cold and warm ischemia time (CIT and WIT, respectively), duration of operation, induction therapy as well as initial maintenance immunosuppressive therapy.

HLA typing was performed using both serological and molecular methods. During the 1990s and early 2000s, serological techniques (complement-dependent cytotoxicity) predominated, identifying broad antigen-level HLA designations (e.g., HLA-DR1, HLA-DR2). Molecular techniques (sequence-specific oligonucleotide probing, sequence-based typing) were progressively implemented from the mid-2000s onward, achieving predominance by 2010 and now providing split-level and allele-level resolution (e.g., DRB1*01:01 vs. DRB1*01:02).

Organ procurement, transplantation and immunosuppression were performed as described previously (9). Induction therapy was administered according to institutional protocol, using either anti-thymocyte globulin (ATG) or IL-2 receptor antagonists (daclizumab or basiliximab). Daclizumab was withdrawn from the market in 2018. At present, basiliximab is administered to all standard kidney transplant recipients and to re-transplant candidates with a PRA level below 85%, while ATG is reserved for highly sensitized recipients (PRA >85%) (Supplementary Figure S1A). Standard immunosuppressive therapy included a calcineurin inhibitor (cyclosporine (CsA) or tacrolimus (Tac), mycophenolic acid, and corticosteroids. CsA was used until 2009 for initial immunosuppressive therapy (Supplementary Figure S1B). For recipients with stable graft function, the recommended target trough concentrations during the first three to four weeks post-transplant were 8–12 ng/mL for tacrolimus (Tac) and 150–200 µg/L for cyclosporine (CsA), with further reductions made based on the institutional protocol. Maintenance immunosuppression was individualized according to clinical course, therapeutic drug monitoring, and adverse events.

HLA mismatches were obtained by Eurotransplant, which program focuses on the HLA-A, -B, and -DR loci. Mismatches for HLA-A and -B are calculated using broad antigen classifications, whereas HLA-DR mismatches are determined based on split antigen definitions regardless of typing methodology (10). This approach may result in slight underestimation of true molecular-level HLA incompatibility in early eras relying predominantly on serological typing. Any bias from mixed typing methodologies would conservatively bias toward the null hypothesis, potentially attenuating rather than inflating observed HLA-DR mismatch associations.

KT recipients were stratified into four groups based on the absence (-, 0 mismatch) or presence (+, ≥1 mismatch) of HLA-DR mismatches, and donor type (SCD or ECD): (1) SCD-DR-, (2) SCD-DR+, (3) ECD-DR- and (4) ECD-DR+.

### Outcome measures

Complications occurring during the first three months after KT were defined as postoperative complications. Initial non-function (INF) of the graft was defined as dialysis dependence or creatinine clearance ≤20 mL/min at three months after KT. Delayed graft function (DGF) was defined as the need for dialysis in the first week post-transplant (11). Kidney allograft biopsies were performed for clinical indications (for-cause strategy) throughout the study period, responding to graft dysfunction signs including elevated serum creatinine (>25% above baseline), new-onset proteinuria, or clinical suspicion of rejection. Graft failure was defined as a return to dialysis or preemptive re-transplantation. Deaths occurring within 30 days after surgery were defined as post-operative deaths.

### Statistical analysis

SPSS software, version 29.0 (SPSS Inc., Chicago, Illinois, USA) and GraphPad Prism software, version 10.4.0 (GraphPad Software, San Diego, California, USA) were used for statistical analysis and graphs. The chi-squared test and the Kruskal–Wallis test were used for analysis of categorical data. For the comparison of continuous variables, the student's *t*-test, analysis of variance (ANOVA) and the Wilcoxon–Mann–Whitney test were used. Uni- and multivariate logistic regression analyses were used to identify independent variables associated with allograft function and morbidity. For multivariate analyses, we employed forward stepwise logistic and Cox regression approaches, including only variables meeting both criteria: (1) clinical relevance based on transplant biology and transplantation literature, (2) statistical significance threshold of *P* < 0.05 in univariate analysis. Graft and patient survival were analyzed using the Kaplan–Meier and the log-rank test, whereas multivariate Cox proportional hazard analysis was applied to assess independent predictors of kidney graft failure. A *P* value < 0.05 was considered as statistically significant. Baseline data are presented as mean values with the standard deviation (SD).

## Results

### Study population

**Figure 1:**
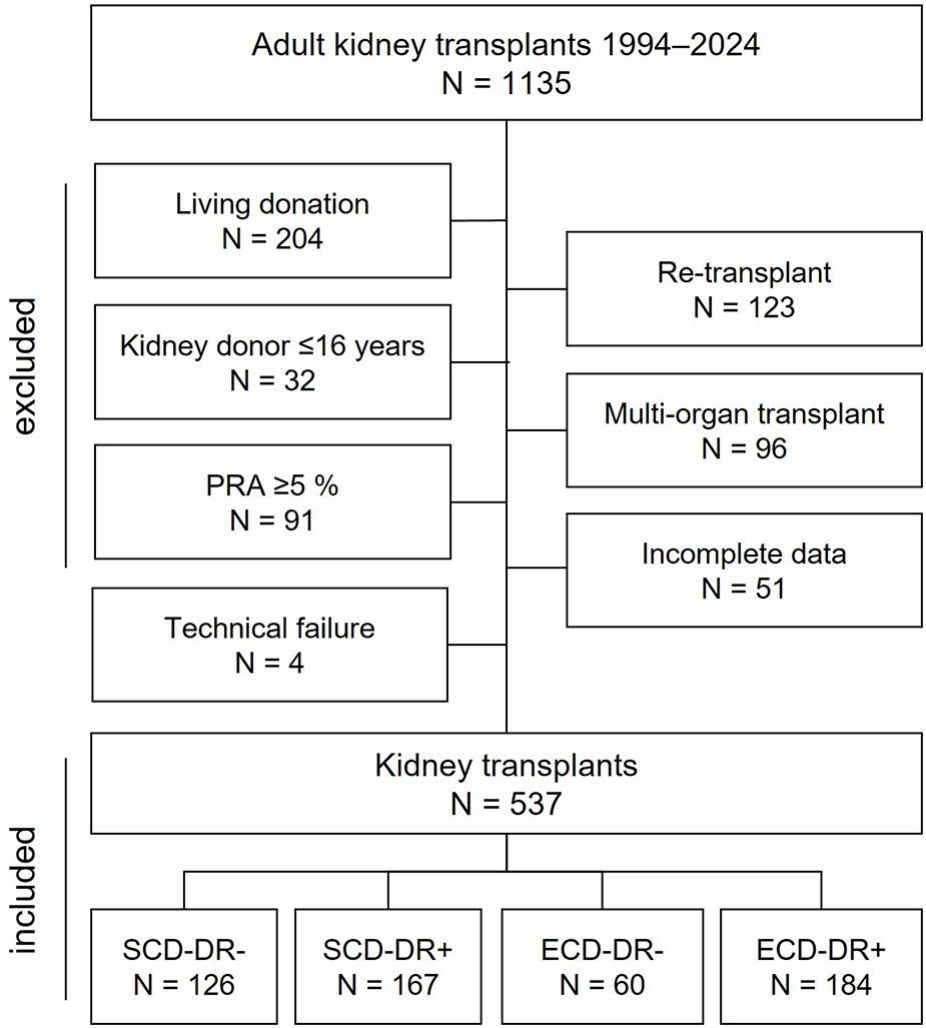
Flow chart showing study population selection. Category numbers do not sum up due to overlap between exclusion criteria.

Between February 1994 and October 2024, 1135 kidney transplants (KT) were performed at our department. 537 KT were included in the study, with 244 (45.4%) kidney grafts from ECDs, and 351 (65.4%) KT having ≥1 HLA-DR mismatches (Figure 1). Mean follow up was 7.3 ± 5.9 years.

**Figure 2:**
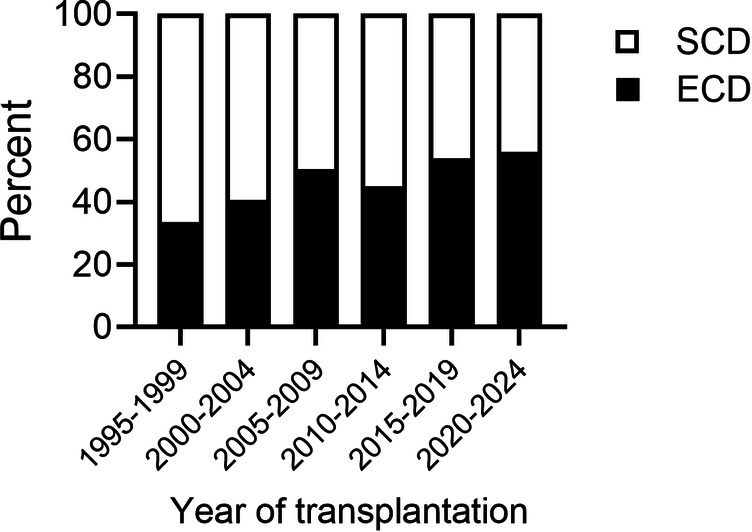
Rates of expended criteria donor (ECD) in primary kidney transplantation at the university hospital of Leipzig between 1995 and 2024.

Figure 2 illustrates the proportion of ECD kidney grafts in primary KT at our transplant center over the last three decades. Comparing the periods from 1995–1999 and 2020–2024, the data reveal a significant increase in ECD KTs from 33.5% to 56.1% (*P* = 0.034).

**Figure 3:**
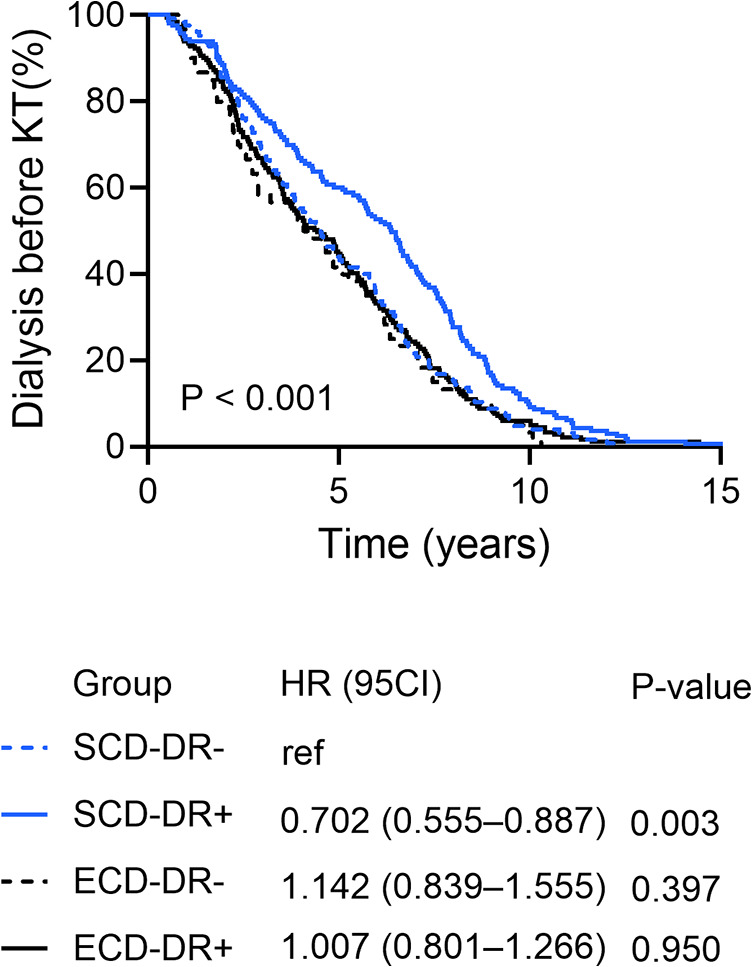
Duration of dialysis prior to KT according to the HLA-DR matching and donor type (standard (SCD) or expended criteria donor (ECD).

The characteristics of the kidney donors, KT recipients, allocation data and transplantation according to the four study groups are summarized in Table 1. In the ECD group, donor age was significantly higher in HLA-DR mismatched KT (ECD-DR− vs. ECD-DR+: 64 ± 6.8 years vs. 67 ± 7.6 years; *P* = 0.003). Patients receiving kidney graft from ECDs were significantly older compared to recipients of kidney grafts from SCDs (48 ± 12.1 years vs. 60 ± 12.3 years; *P* < 0.001) and suffered more often from diabetes mellitus (9.6% vs. 18.9%; *P* = 0.002). Most patients received chronic hemodialysis prior to KT (*N* = 495, 92.2%), without statistical differences between the groups (*P* = 0.294). Duration of dialysis prior to KT was significantly longer in patients receiving SCD-DR + KT compared to those receiving SCD HLA-DR- KT (59.2 ± 33.0 months vs. 72.1 ± 38.7 months; *P* = 0.003), whereas no difference could be observed between the two ECD groups (54.5 ± 32.8 months vs. 58.4 ± 36.7 months; *P* = 0.482) (Figure 3). Regarding transplantation, SCD-DR + KT were associated with a slightly shorter cold ischemia time (CIT SCD-DR- vs. CIT SCD-DR+: 15.4 ± 6.0 h vs. 13.3 ± 5.5 h; *P* = 0.004), whereby warm ischemia time (WIT) was comparable between the groups (*P* > 0.05 each). Mean operation time was longer in the ECD group compared to the SCD group (180 ± 46.5 min vs. 192 ± 55.1 min; *P* = 0.006).

**Table 1:** Donor, recipient, allocation and transplant characteristics of the study population.

Variables	SCD		ECD		*P*-value
	HLA-DR- (*N* = 126)	HLA-DR+ (*N* = 167)	HLA-DR- (*N* = 60)	HLA-DR+ (*N* = 184)	
Donor					
Age, years	40 ± 12.5	41 ± 12.2	64 ± 6.8	67 ± 7.6	<0.001
Gender, male (%)	81 (64.3)	98 (58.7)	25 (41.7)	95 (51.6)	0.016
BMI, kg/m^2^	25.3 ± 4.9	24.6 ± 3.7	26.0 ± 3.5	26.2 ± 4.0	0.001
Cause of death (%)					
CVA	46 (36.5)	65 (38.9)	39 (65.0)	117 (63.6)	<0.001
Hypoxia	12 (9.5)	21 (12.6)	5 (15.0)	12 (6.5)	
Trauma	39 (31.0)	51 (30.5)	9 (20.0)	22 (12.0)	
Infection	3 (2.4)	2 (1.2)	0	1 (0.5)	
Others	26 (20.6)	26 (15.6)	7 (11.7)	32 (17.4)	
Diabetes mellitus (%)	5 (4.0)	10 (6.0)	7 (11.7)	26 (14.1)	0.007
Hypertension (%)	13 (10.3)	22 (13.2)	33 (55.0)	122 (66.3)	<0.001
Recipient					
Age, years	49 ± 11.1	46 ± 12.8	56 ± 14.8	61 ± 11.1	<0.001
Gender, male (%)	77 (61.1)	101 (60.5)	37 (61.7)	125 (67.9)	0.457
BMI, kg/m^2^	25.2 ± 3.9	24.8 ± 4.0	25.2 ± 4.5	26.1 ± 4.2	0.040
Dialysis duration, months	59.2 ± 33.0	72.1 ± 38.7	54.6 ± 32.8	58.4 ± 36.7	<0.001
Cause of ESRD (%)					
Glomerulonephritis	50 (39.7)	75 (44.9)	16 (26.7)	62 (33.7)	0.046
Cystic kidney disease	23 (18.3)	23 (13.8)	15 (25.0)	38 (20.7)	
Interstitial nephritis	16 (12.7)	19 (11.4)	4 (6.7)	14 (7.6)	
Diabetes mellitus	7 (5.6)	6 (3.6)	1 (1.7)	15 (8.2)	
Others	30 (23.8)	44 (26.3)	24 (40.0)	55 (29.9)	
Diabetes mellitus (%)	15 (11.9)	13 (7.8)	5 (8.3)	41 (22.3)	<0.001
Hypertension (%)	116 (92.1)	147 (88.0)	55 (91.7)	171 (92.9)	0.209
Coronary disease (%)	18 (14.3)	20 (12.0)	12 (20.0)	41 (22.3)	0.053
PVD (%)	5 (4.0)	16 (9.6)	3 (5.0)	17 (9.2)	0.207
Allocation					
Gender mismatch	62 (49.2)	79 (47.3)	34 (56.7)	96 (52.2)	0.597
CMV D + R- (%)	28 (22.2)	45 (26.9)	15 (25.0)	37 (20.1)	0.495
No. HLA-mismatches (%)					
0	59 (46.8)	0	18 (30.0)	0	<0.001
1–2	41 (32.5)	36 (21.6)	22 (36.7)	19 (10.3)	
3–4	26 (20.6)	112 (67.1)	20 (33.3)	101 (54.9)	
5–6	0	19 (11.4)	0	64 (34.8)	
HLA-A mismatch	53 (42.1)	127 (76.0)	39 (65.0)	140 (76.1)	<0.001
HLA-B mismatch	59 (46.8)	152 (91.0)	38 (63.3)	173 (94.0)	<0.001
HLA-DR mismatch	0	167 (100)	0	184 (100)	<0.001
Transplant					
Era (%)					
1993–2004	52 (41.3)	73 (43.7)	24 (40.0)	43 (23.4)	<0.001
2005–2014	47 (37.2)	64 (38.3)	30 (50.0)	71 (38.6)	
2015–2024	27 (21.4)	30 (18.0)	6 (10.0)	70 (38.0)	
PRA positive, (%, range)	3 (2.4, 2–3)	5 (3.0, 2–3)	1 (1.7, 3)	9 (4.9, 1–4)	0.512
CIT, hours	15.4 ± 6.0	13.3 ± 5.5	12.0 ± 5.5	11.3 ± 5.0	<0.001
WIT, minutes	44 ± 12.5	43 ± 16.2	45 ± 17.5	44 ± 18.2	0.951
Duration of surgery, minutes	185 ± 47.7	175 ± 45.2	188 ± 43.7	193 ± 58.3	0.015
Induction therapy, ATG/IL2-RA (%)	4 (3.2)/ 32 (25.4)	7 (4.2)/ 48 (28.7)	1 (1.7)/ 22 (36.7)	5 (2.7)/ 99 (53.8)	0.253
Initial immunosuppression (%)					
CNI, Tac/ CsA	76 (60.3)/ 49 (38.9)	97 (58.1)/ 68 (40.7)	37 (61.7)/ 22 (36.7)	140 (76.1)/ 41 (22.3)	0.001
AM drug, AZA/ MMF	11 (8.7)/ 109 (86.5)	13 (7.8)/ 145 (86.8)	3 (5.0)/ 53 (88.3)	5 (2.7)/ 170 (92.4)	0.353
Steroids, prednisolone	122 (96.8)	160 (95.8)	56 (93.3)	178 (96.7)	0.712

Data are shown as mean ± SD. AM, antimetabolite; Aza, azathioprine; ATG, anti-thymocyte globulin; BMI, body mass index; CMV D+/R-, cytomegalovirus status, donor+/recipient-; CIT, cold ischemia time; CNI, calcineurin inhibitor; CsA, Ciclosporin A; CVA, cerebrovascular accident; ECD, expanded criteria donor; ESRD, end-stage renal disease; HLA, human leukocyte antigen; IL2-RA, Interleukin-2 receptor antagonist; PRA, panel reactive antibody; MMF, mycophenolate mofetil; PVD, peripheral vascular disease; SCD standard criteria donor; Tac, tacrolimus; WIT, warm ischemia time.

Immunosuppressive regimens evolved over the observation period but did not differ significantly between SCD and ECD grafts regarding induction strategy (ATG vs. IL-2 RA, *P* = 0.253), antimetabolite choice (MMF vs. AZA, *P* = 0.353), or corticosteroid use (*P* = 0.712). Maintenance CNI type differed across groups (*P* = 0.001 overall), reflecting the institutional transition from cyclosporine to tacrolimus that occurred systematically across all transplant types beginning 2005.

### Postoperative graft function and outcome

The groups were mostly similar regarding the postoperative graft function and rates of surgical complications (Table 2). Time on intensive care unit and hospital stay were comparable between the groups (*P* > 0.05 each).

**Table 2:** Post-operative and long-term outcome parameters. .

Variables	SCD		ECD		*P*-value
	HLA-DR- (*N* = 126)	HLA-DR+ (*N* = 167)	HLA-DR- (*N* = 60)	HLA-DR+ (*N* = 184)	
Kidney function					
Hyperacute rejection (%)	0	1 (0.6)	0	1 (0.5)	0.784
INF (%)	6 (4.8)	11 (6.6)	2 (3.3)	15 (8.2)	0.434
DGF (%)	29 (23.0)	38 (22.8)	13 (21.7)	66 (35.9)	0.010
GFR (mL/ min)					
POD 7	54.7 ± 39.8	50.0 ± 39.4	44.1 ± 32.9	38.8 ± 30.5	0.005
POD 14	78.2 ± 39.0	65.5 ± 39.9	62.0 ± 33.1	50.1 ± 27.8	<0.001
POM 1	92.5 ± 37.9	88.1 ± 36.2	70.6 ± 25.4	63.1 ± 29.8	<0.001
POM 3	96.5 ± 33.2	89.0 ± 35.2	76.4 ± 26.8	68.5 ± 30.6	<0.001
POM 6	99.8 ± 34.2	94.7 ± 35.0	73.5 ± 23.5	71.9 ± 32.8	<0.001
Surgical outcome					
Time on ICU, days	5.8 ± 6.0	4.8 ± 3.5	5.2 ± 3.1	5.8 ± 8.0	0.447
Postoperative complications (%)					
Bleeding/hematoma	15 (11.9)	27 (16.2)	10 (16.7)	39 (21.2)	0.194
Vascular occlusion	3 (2.4)	5 (3.0)	1 (1.7)	9 (4.9)	0.512
Secondary wound healing	26 (20.6)	25 (15.0)	6 (10.0)	42 (22.8)	0.074
Lymphocele	16 (12.7)	13 (7.8)	10 (16.7)	28 (15.2)	0.133
Urine leakage	1 (0.8)	7 (4.2)	1 (1.7)	4 (2.2)	0.281
Hospitalisation, days	26 ± 13.3	26 ± 15.4	27 ± 16.1	30 ± 20.9	0.066
Rejection, 1st year (%)	15 (11.9)	24 (14.4)	6 (10.0)	33 (17.9)	0.325

Data are shown as mean ± SD.

CVA, cerebrovascular accident; DGF, delayed graft function; ECD, expanded criteria donor; GFR, glomerular filtration rate; ICU, intensive care unit; INF, initial non-function; POD, post-operative day; POM, post-operative month; SCD standard criteria donor.

In the ECD group, HLA-DR mismatched KT showed significantly more often a delayed graft function (21.7% vs. 35.9%; *P* = 0.032). In SCD-DR + KT, as well as in the ECD-DR + KT, GFRs were significantly decreased two weeks post-transplant (*P* = 0.030, and *P* = 0.046, respectively).

There were five postoperative deaths. The causes of postoperative death included septic shock (*N* = 1), fulminant pulmonary embolism with cardiac arrest (*N* = 1), cerebral vascular accident (*N* = 2) and acute heart failure (*N* = 1).

### Graft rejection

During the observation period, 282 kidney graft biopsies were performed in 182 patients, with 40.7% undergoing two, 11.5% three, and 3.3% four biopsies. Histopathological analysis revealed graft rejection in 56.5% of the cases.

**Figure 4:**
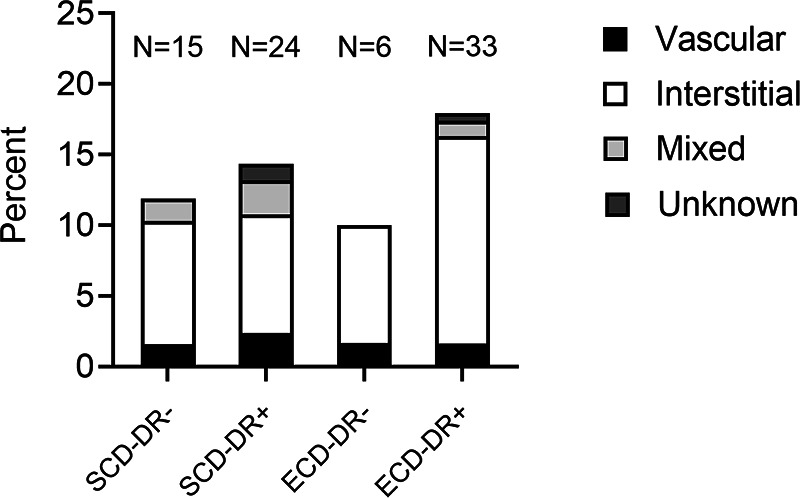
Biopsy-proven graft rejections within the first year after kidney transplantation according to the HLA-DR matching and donor type (standard (SCD) or expended criteria donor (ECD).

In 78 KT recipients (14.5%), rejection arose within the first-year post-transplant. Notably, the incidence of rejection was higher in recipients of ECD-DR + kidney grafts compared to those who received organs from SCDs or ECDs without HLA-DR mismatch. Most cases within the first-year post-transplant presented as interstitial (T cell–mediated) rejections (73.3%–83.3%), while vascular (antibody-mediated) rejections were relatively infrequent across all groups (9.1%–16.7%) (Figure 4).

**Figure 5:**
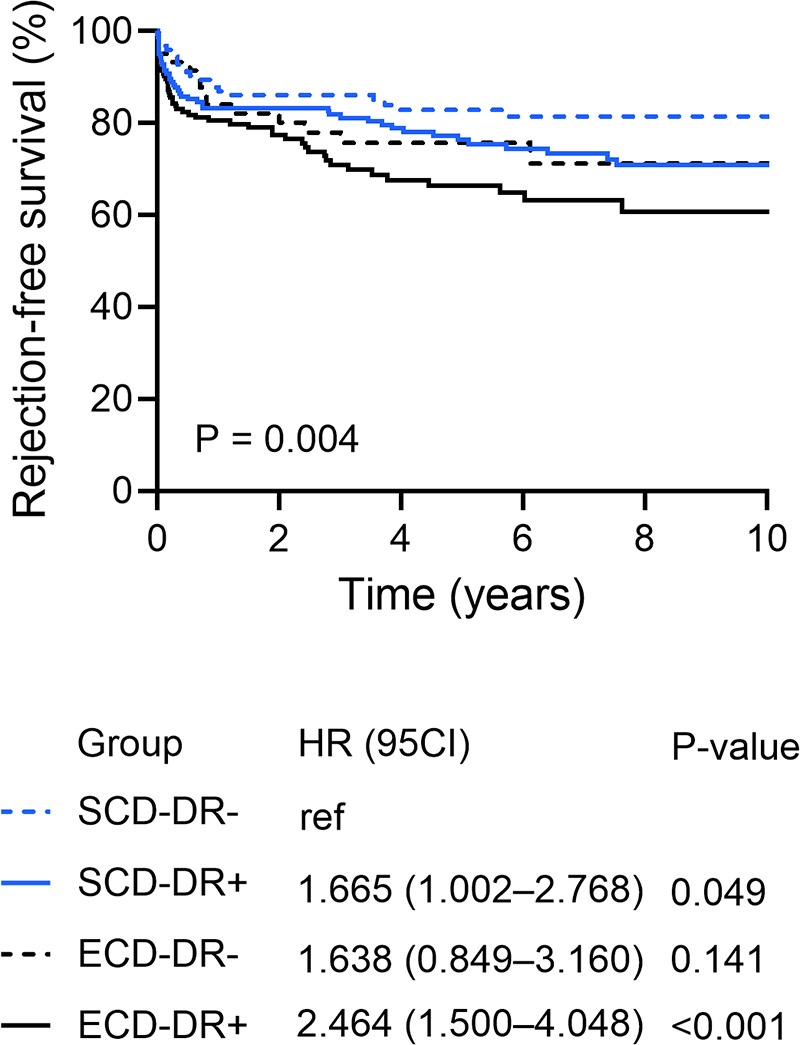
Rejection free survival according to the HLA-DR matching and donor type (standard (SCD) or expended criteria donor (ECD).

During long-term follow-up, rejection-free graft survival was significantly decreased in recipients of ECD-DR + kidneys (reference SCD-DR-: HR 2.464, CI 1.500–4.048; *P* < 0.001) (Figure 5). The incidence density of biopsy-proven rejection was significantly elevated in the ECD-DR + group, reaching 8.2 episodes per 100 patient-years (Table 3), compared to 3.4 episodes per 100 patient-years in the ECD-DR- group (*P* = 0.042). Notably, the incidence of T cell–mediated rejection was significantly higher in ECD-DR + KTs, whereas rates of B cell–mediated rejection did not differ significantly between the groups in subset analysis (Supplementary Figure S2).

**Table 3:** Rates of biopsy-proven rejections per person years of follow-up after kidney transplantation according to the HLA-DR matching and donor type [standard (SCD) or expended criteria donor (ECD)].

Variables	SCD		ECD		*P*-value
	HLA-DR- (*N* = 126)	HLA-DR+ (*N* = 167)	HLA-DR- (*N* = 60)	HLA-DR+ (*N* = 184)	
Person years	1,039.2	1,334.6	355.7	771.3	
No rejections	26	49	12	63	
Incidence density	2.5/ 100	3.7/ 100	3.4/ 100	8.2/ 100	0.005

### Graft survival

**Figure 6:**
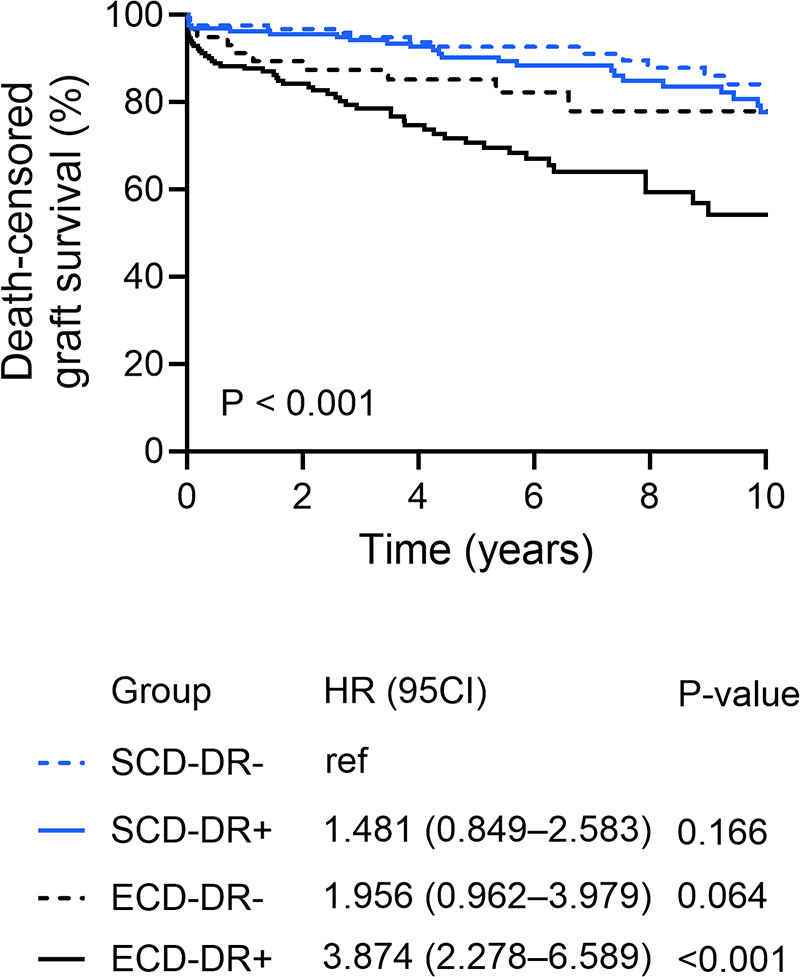
Death-censored graft survival according to the HLA-DR matching and donor type (standard (SCD) or expended criteria donor (ECD).

The mean cumulative death-censored graft survival was 19.0 ± 0.8 years. The 1-, 5-, and 10-year graft survival rates were significantly decreased in ECD KTs compared with SCD KTs (SCD vs. ECD: 96%, 91%, and 80% vs. 88%, 74%, and 61%; *P* < 0.001). Within the ECD cohort, HLA-DR mismatched grafts were associated with poorer graft survival compared to HLA-DR matched KT (1-, 5-, 10-year graft survival: 91%, 85%, and 78% in ECD-DR- vs. 87%, 70%, and 54% in ECD-DR+; *P* = 0.034) (Figure 6). The most frequent causes for graft loss after KT were acute or chronic graft rejection (46 out of 124, 37.1%), chronic allograft nephropathy (26 out of 124, 21.0%), and infection (19 out of 124, 15.3%). Incidence rates of main causes of graft loss are shown in Table 4.

**Table 4:** Rates of different causes of graft loss per person years of follow-up after kidney transplantation according to the HLA-DR matching and donor type [standard (SCD) or expended criteria donor (ECD)].

Variables	SCD		ECD		*P*-value
	HLA-DR- (*N* = 126)	HLA-DR+ (*N* = 167)	HLA-DR- (*N* = 60)	HLA-DR+ (*N* = 184)	
Person years	1,039.2	1,334.6	355.7	771.3	
Cause of graft loss					
Rejection	5	16	5	20	
Incidence density	0.5/ 100	1.2/ 100	1.4/ 100	2.6/ 100	0.006
Chronic allograft nephropathy	4	10	1	11	
Incidence density	0.4/ 100	0.7/ 100	0.3/ 100	1.4/ 100	0.004
Infection	3	3	3	10	
Incidence density	0.3/ 100	0.2/ 100	0.8/ 100	1.3/ 100	0.003

### Patient survival

**Figure 7:**
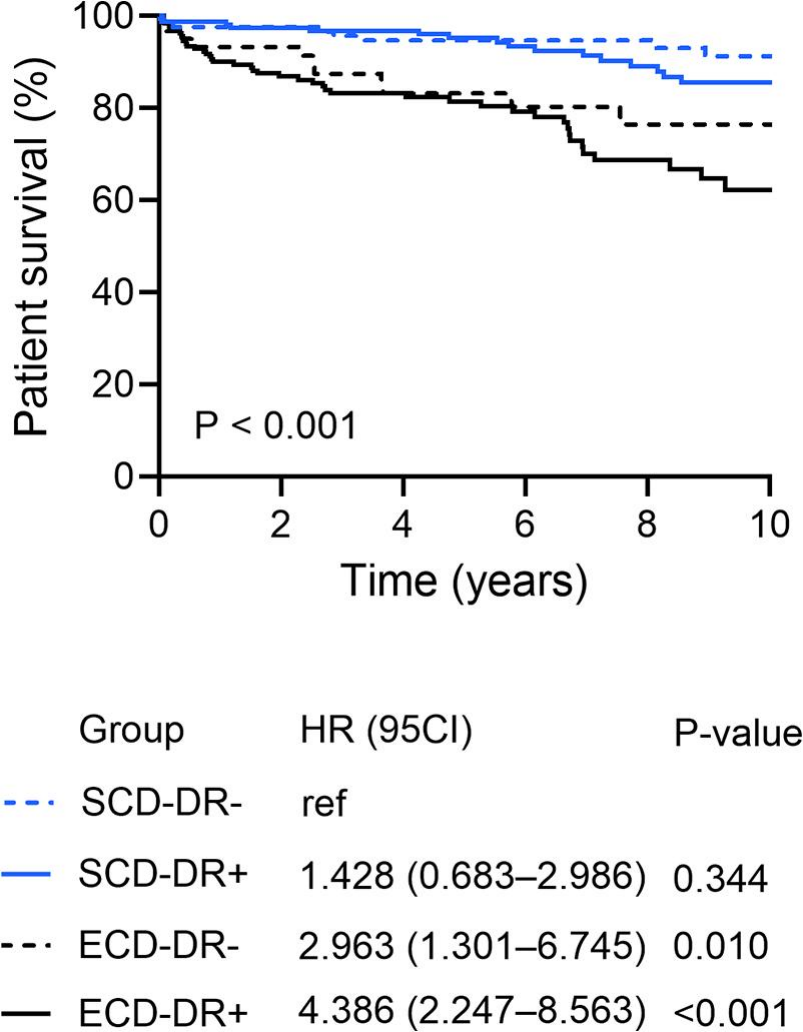
Patient survival according to the HLA-DR matching and donor type (standard (SCD) or expended criteria donor (ECD).

Patient survival rates at 1, 5, and 10 years were significantly lower in recipients of ECD kidneys compared to SCD grafts (SCD vs. ECD: 98%, 95%, and 88% vs. 91%, 82%, and 66%; *P* < 0.001). However, HLA-DR mismatch did not appear to affect overall patient survival (1-, 5-, 10-year graft survival: 93%, 83%, and 76% in ECD-DR- vs. 90%, 81%, and 62% in ECD-DR+; *P* = 0.246) (Figure 7). The most common causes of death after KT were fulminant sepsis (24 out of 9, 26.7%) and cardiac events (14 out of 90, 15.6%).

### Multivariate analyses

Multivariate regression analysis of risk factors of delayed graft function, rejection, graft and patient survival are shown in Table 5.

**Table 5:** Multivariate regression analysis of predictors of delayed graft function, rejection, kidney graft loss and KT recipient mortality.

Variables	Delayed graft function (1)				Rejection (2)				Graft failure (death-censored) (2)				Patient survival (2)			
	UVA		Multivariate analysis		UVA		Multivariate analysis		UVA		Multivariate analysis		UVA		Multivariate analysis	
	*P*-value	OR	95% CI	*P*-value	*P*-value	HR	95% CI	*P*-value	*P*-value	HR	95% CI	*P*-value	*P*-value	HR	95% CI	*P*-value
Donor																
Age	0.056				0.012	NS	NS	NS	<0.001	NS	NS	NS	<0.001	NS	NS	NS
Gender, male	0.329				0.786				0.123				0.007	NS	NS	NS
BMI	0.079				0.583				0.163				0.864			
Cause of death																
CVA vs. non-CVA	0.662				0.377				0.052				0.038	NS	NS	NS
Diabetes mellitus	0.792				0.185				0.211				0.107			
Hypertension	0.062				0.090				<0.001	NS	NS	NS	<0.001	NS	NS	NS
ECD	0.012	NS	NS	NS	0.005	NS	NS	NS	<0.001	NS	NS	NS	<0.001	2.286	1.312–3.984	0.004
Recipient																
Age	0.025	NS	NS	NS	0.728				0.310				<0.001	1.067	1.040–1.096	<0.001
Gender, male	0.176				0.246				0.068				0.159			
BMI																
18.5–24.9 (ref)	0.002			NS	0.805				0.038			NS	0.846			
<18.5	0.256	NS	NS	NS	0.660				0.414	NS	NS	NS	0.410			
25–29.9	0.830	NS	NS	NS	0.568				0.768	NS	NS	NS	0.777			
>30	<0.001	NS	NS	NS	0.368				0.006	NS	NS	NS	0.997			
Dialysis duration	0.008	1.008	1.001–1.014	0.023	0.739				0.126				0.027	NS	NS	NS
Cause of ESRD																
GN vs. non-GN	0.889				0.190				0.356				0.146			
Diabetes mellitus	0.197				0.004	1.910	1.228–2.971	0.004	<0.001	1.825	1.118–2.976	0.016	<0.001	1.913	1.119–3.271	0.018
Hypertension	0.860				0.018	NS	NS	NS	<0.001	2.375	1.331–4.238	0.003	0.225			
Coronary disease	0.245				0.368				0.542				<0.001	NS	NS	NS
PVD	0.915				0.411				0.881				0.011	NS	NS	NS
Allocation																
Gender mismatch	0.168				0.063				0.020	NS	NS	NS	0.036	NS	NS	NS
CMV D + R-	0.739				0.179				0.856				0.586			
HLA-ABDR mismatch																
0 (ref)	0.707				0.446				0.440				0.015			NS
1–2	0.724				0.141				0.656				0.075	NS	NS	NS
3–4	0.678				0.130				0.264				0.019	NS	NS	NS
5–6	0.274				0.167				0.151				0.002	NS	NS	NS
HLA-A mismatch	0.921				0.667				0.496				0.060			
HLA-B mismatch	0.888				0.047	NS	NS	NS	0.155				0.056			
HLA-DR mismatch	0.061				0.006	NS	NS	NS	0.002	NS	NS	NS	0.025	NS	NS	NS
SCD HLA-DR- (ref)	0.011			NS	0.004			0.003	<0.001			<0.001	<0.001			NS
SCD HLA-DR + s	0.975	NS	NS	NS	0.049	1.733	1.032–2.910	0.038	0.166	1.302	0.718–2.369	0.386	0.344	NS	NS	NS
ECD HLA-DR-	0.837	NS	NS	NS	0.141	1.829	0.941–3.553	0.075	0.064	2.013	0.958–4.237	0.065	0.010	NS	NS	NS
ECD HLA-DR+	0.012	NS	NS	NS	<0.001	2.631	1.578–4.389	<0.001	<0.001	3.528	2.008–6.197	<0.001	<0.001	NS	NS	NS
Transplant																
Era	0.151				0.185				0.468				0.645			
PRA positive	0.861				0.552				0.908				0.599			
CIT	0.879				0.840				0.913				0.004	NS	NS	NS
WIT	0.016	1.019	1.004–1.033	0.011	0.560				0.176				0.089			
Duration of surgery	0.026	NS	NS	NS	0.980				0.016	NS	NS	NS	0.527			
Induction therapy	0.152				0.570				0.785				0.157			
Initial immunosuppression																
Tac vs. CsA	0.039	NS	NS	NS	0.021	1.801	1.258–2.579	0.001	0.120				0.307			
AM	0.609				0.459				0.762				0.926			
Prednisolone	0.885				0.738				0.898				0.770			
Kidney function																
DGF	N/A	N/A	N/A	N/A	0.359				0.392				0.069			
Rejection, 1st year	N/A	N/A	N/A	N/A	N/A	N/A	N/A	N/A	<0.001	2.948	1.991–4.381	<0.001	0.075			

Following variables were tested in univariate analysis but failed to show significancy: antimetabolites (mycophenolate mofetil), steroids (prednisolone).

95% CI, 95% confidence interval; AM, antimetabolite BMI, body mass index; CIT, cold ischemia time; CMV D+/R, cytomegalovirus status, donor+/recipient-; CsA, Ciclosporin A; CVA, cerebrovascular accident; DGF, delayed graft function; ECD, expanded criteria donor; era, transplant era (1995–2004 vs. 2005–2014 vs. 2015–2024); ESRD, end-stage renal disease; HLA, human leukocyte antigen; HR, hazard ratio; NS, not significant; PRA, panel reactive antibody; SCD standard criteria donor; UVA, univariate analysis; Tac, tacrolimus; WIT, warm ischemia time. (1) linear regression, (2) Cox regression.

### Delayed graft function

Independent predictors of delayed graft function (DGF) were duration of dialysis before KT [adjusted HR (aHR) 1.008; *P* = 0.023] and WIT (aHR 1.019; *P* = 0.011). In multivariate regression analysis, neither ECD nor HLA-DR were independent predictors of DGF.

### Graft rejection

After adjustment for important covariates using the Cox proportional hazards model, HLA-DR mismatch remained an independent predictor of graft rejection in both SCD KT (aHR 1.733; *P* = 0.038) and ECD KT (aHR 2.631; *P* < 0.001), compared to SCD-DR matched KT. Other independent predictors of graft rejection were recipient diabetes mellitus (aHR 1.910; *P* = 0.004) and initial immunosuppression with ciclosporin instead of tacrolimus (aHR 1.801; *P* = 0.001).

### Graft survival

In multivariate Cox regression analysis recipient diabetes mellitus (aHR 1.825; *P* = 0.016), recipient hypertension (aHR 2.375; *P* = 0.003), HLA-DR mismatch in ECD KT (aHR 3.528; *P* < 0.001), and graft rejection within the first year after KT (aHR 2.948; *P* < 0.001) remained independent predictors of graft failure.

### Patient survival

ECD (aHR 2.286; *P* = 0.004), recipient age (aHR 1.067; *P* < 0.001), and recipient diabetes mellitus (aHR 1.913; *P* = 0.018), were significantly associated with patient survival in multivariate Cox regression analysis. HLA-DR mismatch showed no influence on mortality rates after primary KT.

CIT (analyzed continuously and using 12- and 18-hours cutoffs), induction therapy, antimetabolite choice, and corticosteroids demonstrated no independent influence on any outcome when adjusted for era and other covariates.

Subdividing HLA-A, -B, and -DR mismatch rates into 0, 1, or 2 mismatches did not alter the regression analysis results.

### Sensitivity analyses

#### Transplant era and immunosuppression

To address concerns regarding potential confounding by evolving immunosuppressive protocols and HLA typing methodologies, we performed sensitivity analyses restricted to the transplant eras.

Era-stratified analyses demonstrated that HLA-DR mismatch effects on graft rejection were consistent across first two decades examined (1995–2004, 2005–2014), with ECD-DR+ consistently showing significantly increased rejection risk compared to SCD-DR− (all *P* < 0.05) (Supplementary Figure S3A). Stratified analysis in the recent era (2015–2024) was limited by small subgroup sample sizes (SCD-DR−, *N* = 27) and minimal early graft loss events, resulting in unstable hazard ratio estimates (>1,000). This limitation reflects insufficient event density in recent cohorts with limited long-term follow-up rather than absence of HLA-DR effects. In multivariate models including transplant decade as a categorical covariate, era adjustment had no independent influence on DGF, rejection, graft loss, or patient survival outcomes. Sensitivity analyses restricted to the modern era (2010–2024), during which tacrolimus-based maintenance immunosuppression and molecular HLA typing methodologies were standard, demonstrated consistent HLA-DR mismatch effects in the modern-era cohort, with significantly higher rates of graft loss in the ECD-DR + KT group compared to SCD-DR- KT (Supplementary Figure S3B).

### Delayed graft function

Univariate Cox regression analyses of graft rejection and graft survival across HLA-DR groups, stratified by the presence or absence of DGF revealed that ECD-DR+ recipients exhibited significantly higher rates of rejection and graft loss compared to SCD-DR- recipients, independent of the presence of DGF (Supplementary Figure S4). The higher DGF rates in ECD-DR+ recipients (35.9% vs. 21.7%, *P* = 0.032) likely reflect differential organ quality rather than serving as an independent determinant of long-term graft failure. The association between HLA-DR mismatch and graft loss appears to operate primarily through *T* cell–mediated rejection mechanisms, which were significantly elevated in ECD-DR+ recipients (8.2 vs. 3.4 episodes per 100 patient-years, *P* = 0.042), rather than through DGF-related pathways.

## Discussion

The proportion of kidney transplants (KT) from extended criteria donors (ECDs) has increased significantly over the past decades, and this trend is likely to result in more younger recipients receiving ECD grafts in the future.

Our study shows that in ECD KT, HLA-DR mismatches are associated with higher rejection rates and reduced graft survival. These findings challenge the assumption that immunologic matching is less critical for ECD grafts and suggest that optimizing HLA-DR compatibility—particularly for younger or lower-risk recipients—may be beneficial (6).

The percentage of 5–6 HLA mismatches was higher in the ECD-DR+ group than in the SCD-DR+ group (11.4% vs. 34.8%). This likely reflects the greater polymorphism of HLA-A and -B compared to HLA-DR, as well as the lower prioritization of mismatches within the ESP. However, statistical analyses showed no predictive value for either the total number of HLA mismatches or HLA-A and -B mismatches in transplant outcomes. Notably, in our cohort, only eight KTs with an HLA-DR mismatch were matched for HLA-A and -B (four in SCD-DR+ and four in ECD-DR+), preventing further analysis.

### Rejection

This study demonstrates that HLA-DR mismatches are an independent risk factor for graft rejection in both SCD and ECD KTs. The likelihood of T cell-mediated graft rejection (TCMR) was particularly increased in kidneys from ECDs. A previous retrospective study by Halleck et al. analyzing 972 KTs from both living and deceased donors also identified HLA-DR mismatches as independent risk factors for TCMR. In elderly KT recipients, HLA-DR mismatches were associated with a significantly higher incidence of TCMR and the development of *de novo* donor-specific antibodies, leading to poorer graft survival. Most TCMR episodes occurred within the first six months post-transplant. The incidence of antibody-mediated rejection was similar between HLA-DR mismatch-positive and -negative transplants (5%–9% within 7 years post-transplant), consistent with our findings (12).

In the Eurotransplant region, ECD kidneys are primarily allocated to older recipients. However, the impact of immunosenescence on KT outcomes is not fully understood. While advancing age in recipients is associated with a reduced incidence of allograft rejection, likely due to diminished T cell–mediated immune responses, ECD grafts are known to carry a higher risk of both acute and chronic rejection (12–15). This suggests that HLA-DR mismatches may disrupt the balance between the reduced immune activity in elderly recipients and the heightened immunogenicity of ECD grafts (16).

### Graft survival

In the present study, HLA-DR mismatch was an independent predictor of graft loss in ECD KT compared with SCD-matched KT. This aligns with de Fijter et al., who reported that HLA-DR matching improves five-year graft survival and function in ESP kidney transplants (8). However, in this study, the impact of HLA-DR mismatch was examined only in KTs from donors and recipients over 65. In contrast, our study defined organ quality based on both donor age and medical history. The increased risk of graft failure in ECD recipients is likely multifactorial. ECD kidneys were mostly transplanted into older recipients with more comorbidities, potentially exacerbating graft vulnerability and increasing the risk of repeated rejection and graft loss. It is plausible that SCD kidneys are better able to compensate for the negative effects of HLA-DR mismatches. In a retrospective analysis of over 44,000 living donor kidney transplants, Budhiraja et al. showed that higher donor eGFR significantly reduced the risk of graft loss in recipients with HLA-DR mismatches, but not in those with HLA-DR matches. This suggests that superior donor kidney function can partially offset the immunological burden of an HLA-DR mismatch. The effect was most pronounced in younger recipients (aged 18–39), in whom HLA-DR compatibility not only improved long-term graft survival but also reduced the risk of sensitization—a key consideration, as younger patients are more likely to require multiple transplants and are at higher risk of developing *de novo* HLA antibodies (7). In our study, the small number of younger recipients precluded statistical analysis, and data on *de novo* sensitization were limited.

### Limitations

This study has several limitations. First, its retrospective, single-center, and non-randomized design may introduce bias.

Second, the extended investigation period constrained data evaluation, highlighting the need for controlled, prospective studies. Detailed assessment of graft rejection grades was restricted by potential inconsistencies in histopathological evaluation over time (revisions in the Banff classification), and era-dependent confounding factors—such as changes in medical care, and immunosuppression—cannot be fully excluded. Heterogeneous biopsy reporting practices and potential missing data across eras may introduce residual confounding, particularly when interpreting long-term graft survival differences and the mechanisms of rejection. Biopsy diagnoses were therefore limited to categorical assessment (*T* cell–mediated vs. antibody-mediated rejection), precluding more granular immunological interpretation. The for-cause biopsy approach may introduce differential surveillance bias, potentially favoring detection of rejection in recipients with more clinical graft dysfunction (possibly overrepresented in ECD-DR+ group).

Third, our analysis relies on low-resolution antigen-level HLA typing, which represents a significant limitation in the context of contemporary immunogenetics. Recent evidence demonstrates that eplet-level and molecular HLA mismatch provide superior predictive value for *de novo* donor-specific antibodies, *T* cell–mediated rejection incidence, and long-term graft survival compared to traditional antigen-level assessment (17–19). The consistent association between HLA-DR antigen-level mismatch and adverse outcomes observed in our cohort, despite this methodological limitation, suggests that the true immunological impact at the molecular/eplet level may be even more pronounced. This finding provides additional justification for implementation of extended genomic HLA typing in ECD allocation algorithms, particularly when considering high-immunogenicity recipients. Future prospective studies should employ extended genomic HLA typing to define HLA-DR mismatches at the allelic and eplet levels, which would provide mechanistic insights into *T* cell vs. antibody-mediated rejection pathways and enable more precise risk stratification for personalized allocation strategies.

Fourth, this study enrolled a selected population with low baseline alloimmunological risk (PRA ≤5% at transplant), which restricts generalizability to highly sensitized patients (PRA >5%). Sensitized recipients represent a distinct immunological phenotype with higher baseline rejection risk and potential pre-existing HLA-specific memory responses that may differentially modulate the effect of HLA-DR mismatch. The cohort composition limits applicability to sensitized kidney transplant candidates, in whom the magnitude of HLA-DR mismatch effects may differ.

Fifth, the absence of comprehensive *de novo* donor-specific antibody (dnDSA) monitoring throughout the follow-up period limits mechanistic interpretation of HLA-DR mismatch effects. Our histological data suggest *T* cell–mediated rejection predominates (73.3–83.3% of first-year rejection episodes), but we cannot quantify the contribution of antibody-mediated mechanisms or subclinical DSA development. Prospective studies incorporating serial dnDSA monitoring would elucidate whether HLA-DR mismatch primarily operates through *T* cell–mediated rejection or antibody-mediated pathways, and whether sensitized recipients demonstrate qualitatively different mechanisms of HLA-DR mismatch-related graft loss. Prospective studies incorporating serial dnDSA monitoring would elucidate whether HLA-DR mismatch primarily operates through *T* cell –mediated rejection or antibody-mediated pathways.

## Conclusion

In conclusion, due to the elevated rejection rates and impaired graft survival observed, ECD kidney grafts with HLA-DR mismatches warrant careful consideration during organ allocation. However, prospective multicenter validation is essential before implementing policy changes. If these findings are confirmed in controlled prospective studies, prioritizing HLA-DR matching—including high-resolution and eplet-level matching—could reduce rejection risk and improve graft longevity in this population. At the same time, the benefits of HLA compatibility must be weighed against the potential risks of prolonged waiting times, which may offset these advantages.

## Data Availability

The data analyzed in this study is subject to the following licenses/restrictions: Our database contains highly sensible data which may provide insight in clinical and personnel information about our patients and lead to identification of these patients. Therefore, according to organizational restrictions and regulations these data cannot be made publicly available. However, the datasets used and/or analyzed during the current study are available from the corresponding author on reasonable request. Requests to access these datasets should be directed to uwe.scheuermann@medizin.uni-leipzig.de.

## Supplementary material

Supplementary Figure S1 **(A)** Induction therapy and **(B)** type of calcineurin inhibitor used for maintenance immunosuppressive therapy in primary kidney transplantation at the University Hospital of Leipzig between 1995 and 2024. ATG, anti-thymocyte globulin; CsA, ciclosporin A; m-TORi, mammalian target of rapamycin-inhibitor (everolimus or sirolimus); Tac, tacrolimus.

Supplementary Figure S2 Rejection free survival according to the HLA-DR matching and donor type (standard (SCD) or expended criteria donor (ECD). **(A)** *T* cell mediated rejections, **(B)** B cell mediated rejections.

Supplementary Figure S3 Univariate Cox regression analyses of graft rejection and graft loss across HLA-DR groups, stratified by (A) transplant eras (1995–2004, 2005–2014, and 2015–2024) and (B) modern transplant era (2010–2024, tacrolimus-era with molecular HLA typing standards). Due to the small number of cases within the subgroups and the limited post-transplant follow-up period, an analysis of the 2015–2024 transplant era with respect to the hazard ratio for graft survival could not be conducted.

Supplementary Figure S4 Univariate Cox regression analyses of graft rejection and graft loss across HLA-DR groups, stratified by the presence or absence of delayed graft function (DGF).
