# Supplementary figures and images for: Impaired graft survival in kidney transplants from expanded criteria donors with HLA-DR mismatch
Source: Front Transplant. 2026 Feb 12;5:1733351. doi: 10.3389/frtra.2026.1733351 (PMC12936035; doi:10.3389/frtra.2026.1733351)

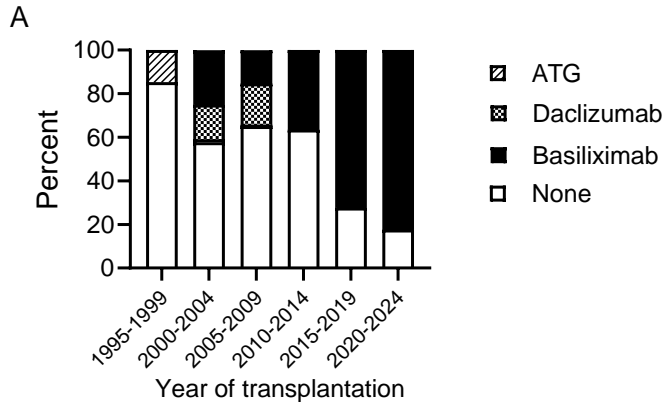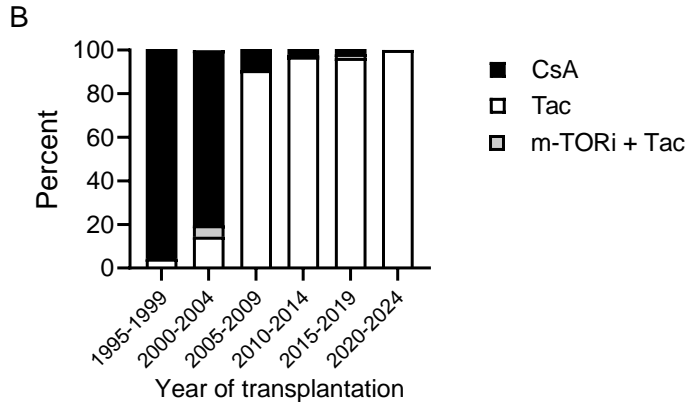

Supplement: Supplementary Figure S1 — (A) Induction therapy and (B) type of calcineurin inhibitor used for maintenance immunosuppressive therapy in primary kidney transplantation at the University Hospital of Leipzig between 1995 and 2024. ATG, anti-thymocyte globulin; CsA, ciclosporin A; m-TORi, mammalian target of rapamycin-inhibitor (everolimus or sirolimus); Tac, tacrolimus. [file Datasheet1.pdf]

A

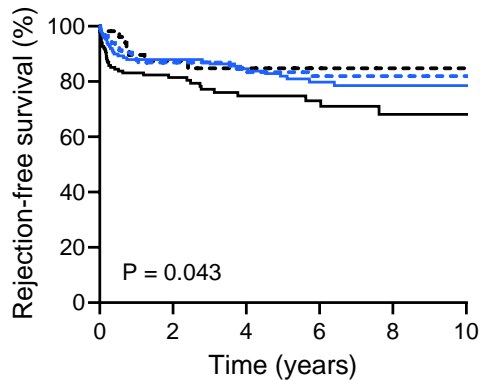

| Group       | HR (95CI)           | P-value |
|-------------|---------------------|---------|
| --- SCD-DR- | ref                 |         |
| — SCD-DR+   | 1.182 (0.663–2.108) | 0.571   |
| --- ECD-DR- | 0.875 (0.368–2.082) | 0.763   |
| — ECD-DR+   | 1.884 (1.089–3.259) | 0.024   |

B

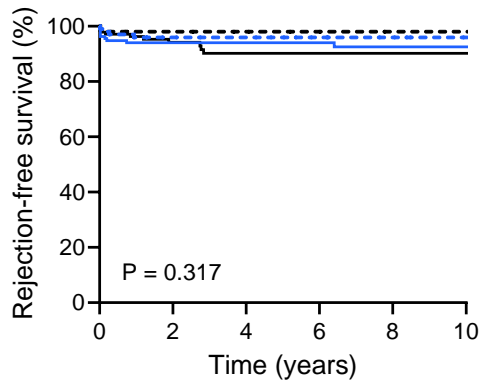

| Group       | HR (95CI)           | P-value |
|-------------|---------------------|---------|
| --- SCD-DR- | ref                 |         |
| — SCD-DR+   | 2.166 (0.689–6.802) | 0.186   |
| --- ECD-DR- | 1.280 (0.233–7.024) | 0.141   |
| — ECD-DR+   | 2.700 (0.850–8.577) | 0.092   |

Supplement: Supplementary Figure S2 — Rejection free survival according to the HLA-DR matching and donor type (standard (SCD) or expended criteria donor (ECD). (A) T cell mediated rejections, (B) B cell mediated rejections. [file Datasheet2.pdf]

A

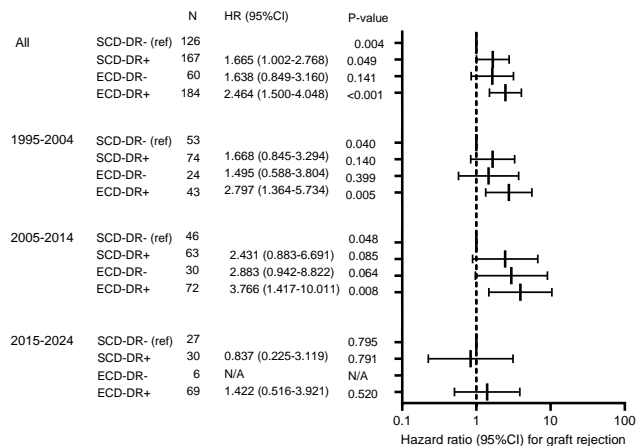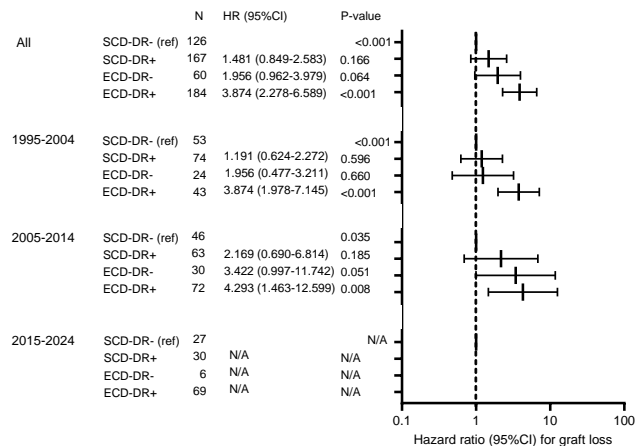

B

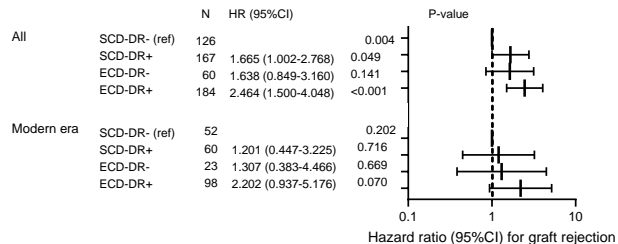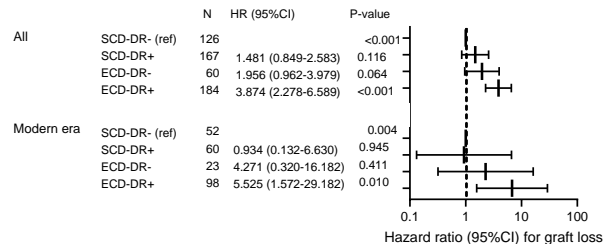

Supplement: Supplementary Figure S3 — Univariate Cox regression analyses of graft rejection and graft loss across HLA-DR groups, stratified by (A) transplant eras (1995–2004, 2005–2014, and 2015–2024) and (B) modern transplant era (2010–2024, tacrolimus-era with molecular HLA typing standards). Due to the small number of cases within the subgroups and the limited post-transplant follow-up period, an analysis of the 2015–2024 transplant era with respect to the hazard ratio for graft survival could not be conducted. [file Datasheet3.pdf]

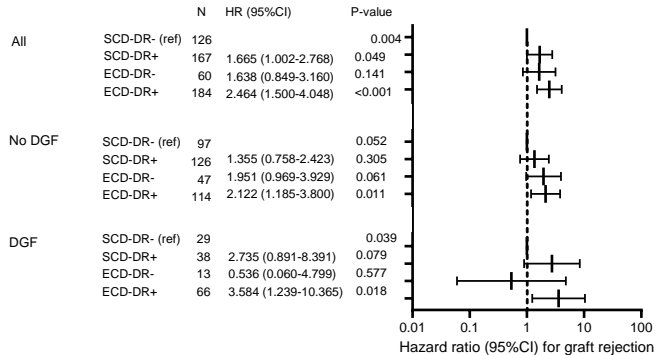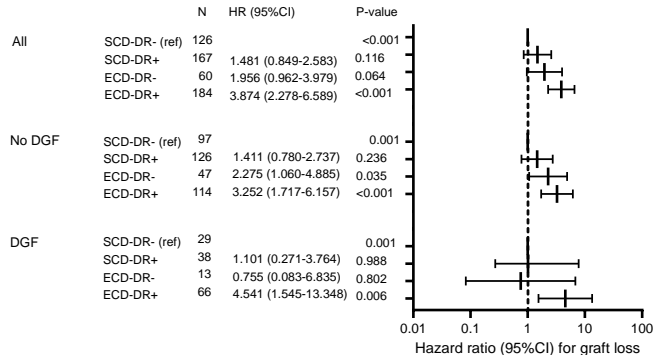

Supplement: Supplementary Figure S4 — Univariate Cox regression analyses of graft rejection and graft loss across HLA-DR groups, stratified by the presence or absence of delayed graft function (DGF). [file Datasheet4.pdf]
